# Iranian homograft tissue processing

**DOI:** 10.21542/gcsp.2016.7

**Published:** 2016-03-31

**Authors:** Alireza Heidary Rouchi, Seyed Amirhosein Tavakoli, Mitra Mahdavi-Mazdeh

**Affiliations:** Iranian Tissue Bank and Research Center, Tehran University of Medical Sciences, Tehran, Iran

**Keywords:** Tissue Procurement, Tissue Transplantation, Heart Valve, Tissue Processing, Allograft, Quality Assurance

## Abstract

Tissue transplantation is a life-enhancing therapeutic modality for damaged or non-functioning tissues. In most cases, there is no alternative other than human tissue as replacement, and taking into account the ever-increasing demand for tissue grafts, it makes sense to set up an establishment in charge of human tissue procurement to meet local needs. A quality assurance system, clearly defined standards, and regular audits complement the infrastructure which make this activity feasible. The process of tissue procurement consists of donor identification, consent, tissue recovery, donor screening and testing, tissue processing, preservation, packaging, labeling, terminal sterilization, storage and distribution. The transplantation of homograft heart valves remains controversial, due to the availability of prosthetic and bioprosthetic alternatives. The limited durability of homografts has not yet outweighed the advantages which this graft offers. Adherence to regulations and regularly revised guidelines improve long-term efficacy and minimizes complications or malfunction. Furthermore, the lower price of homograft heart valves and the removal of the need for a lifetime of anticoagulation therapy are noteworthy advantages of this replacement. In our practice, the proportion of homograft heart valves meeting release criteria and successfully implanted grafts were 83% and 95%, respectively.

## Background

Allogenic grafts – or allografts – are tissue grafts to replace damaged or non-functioning tissues in order to restore normal function. Prosthetic replacements have certain advantages, but the greater biocompatibility, better performance, and fewer complications of tissue grafts have made them the replacement of choice for some surgeons. Nevertheless, the safety and efficacy of procured tissues is of enormous importance.

The first experience in the field of tissue transplantation saw a fresh aortic valve placed into the descending aorta and was performed by Gordon Murray in 1956^1^. Afterwards, Donald Ross, in 1962, was the pioneering surgeon whom carried out the first homograft implantation in the cardiac position^2^. Thereafter, prosthetic heart valves were launched and used in the 1960s before xenografts came along in the 1970s^3^.

Human heart valves are the preferred replacement in right and left outflow tract reconstruction^4^. Patients with small aortic roots, or aortic root endocarditis, benefit most from homograft implantation^5^. Homografts are also preferred to prosthetic or bioprosthetic heart valves for patients for whom long-term anticoagulation therapy is complicated, or even contraindicated, such as children, women of child-bearing age and adults with physically demanding occupations.

Compared with mechanical valves, allografts have better hemodynamic performance, low thromboembolic rates without anticoagulation, no mechanical damage to blood cells, and low frequency of post-operative endocardititis, but limited durability^3,6,7,8^. Considering the pros and cons of homografts, these tissue valves are more cost-benefit and affordable for a majority of settings worldwide. This fact has led to increasing demand for aortic and pulmonary valve homografts. To supply enough, safe, efficient and durable homografts requires a standard system run based on Standard Operating Procedures (SOPs)^9^.

To tackle this requirement, we try to comprehensively review the general concepts of tissue procurement, where particular emphasis is given to quality, safety and heart valve processing as the most biocompatible and affordable replacement for valvular dysfunction, once valve replacement is indicated. This article aims to systematically summarize the procedures of human tissue procurement, to show the feasibility of this activity, in light of ever-increasing need^10^.

## Tissue procurement intended for transplantation

### Background information

“Human cell and tissue transplantation can save lives or restore essential functions where no alternatives of comparable effectiveness exist”^11^. Compared with organ transplantation, tissue donation and transplantation is an activity with a wider spectrum of applicability, and one which more patients benefit from its life-saving, or life-improving nature^12^. Wider indications and more patients suffering from tissue impairment have caused a rapid growth in the need for human tissues. Unlike organ donation, most tissue donors are non-heart-beating deceased donors from whom tissues can be retrieved within 24 hours of death. We therefore have a large pool of tissue donors and no concerns around tissue shortage.

## Human tissue procurement

**Figure 1.**
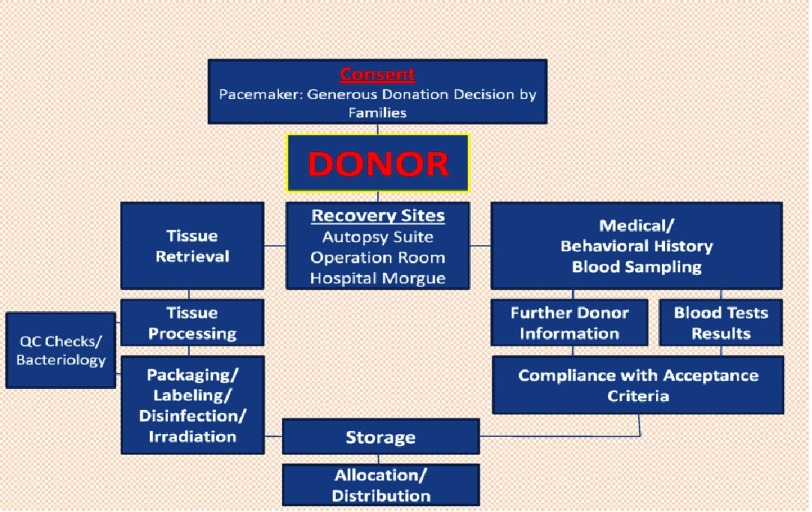
Donation to Implantation Process.

The donation-to-implantation process is illustrated schematically in Figure 1. Speed is governed by the decisions of families who respond positively once given the option of donation. Following donor identification,n and obtaining family consent, the screening of medical, social and behavioral history of the potential donor begins. General assessments and physical exams are performed and blood sampling for testing is taken. Afterwards, and following the first level of assurance concerning donor eligibility, tissue retrieval is performed based on consented tissues for donation. Tissue procurement then follows this typical sequence:

- Tissue recovery: Donor selection, locating donor, obtaining consent, screening medical and behavioral history, tissue retrieval, tissue sampling for microbiological studies (culturing).
- Testing: Serological and microbiological testing, tissue specific testing.
- Processing: Cleaning, cutting, trimming, sizing, mechanical and chemical treatment (antibiotic decontamination/ incubation), culturing (filter culturing, tissue sample, and swabbing culturing) prior and post antibiotic incubation, preservation, controlled rate freezing.
- Packaging: Final sterilization for musculoskeletal tissues, storage, distribution.

### Quality assurance programs

To procure safe and efficient tissues, quality control dominates the whole process from donor selection to the post-implantation phase. The programs which guarantee the quality and safety of the procured tissues must rely on comprehensive Standard Operating Procedures (SOPs)^9^. Internal periodic assessments and audit programs by accreditation associations are crucial strategies to maintain responsible tissue procurement. There are different ways to address the necessity for a quality system:

- International Standards Organization (ISO).
- Good Manufacturing Practice (GMP): A set of regulations and guidelines, or the quality assurance scheme, which ensures that the final product is produced and controlled in accordance with the quality standards.
- Good Tissue Practice (GTP): The United States Food and Drug Administration (FDA) implemented a regulation which includes the methods, facilities and controls used for the manufacture of human tissue and cell products. The aim of these GTPs is to prevent the introduction, transmission and spread of communicable diseases through cellular and tissue-based products^9^.

### Traceability

Traceability refers to the ability to locate and identify tissues/cells at any stage from procurement, through processing, testing and storage, to distribution to the recipient, or disposal using a unique coding system. It also implies the ability to identify the donor, tissue establishment and recipients.

Traceability also covers the ability to locate and identify all relevant data related to products and materials coming into contact with those tissues/cells. It also makes the long-term follow up of tissues possible. A “Feedback Report Form” is sent with the allograft to the recipient center. In this form, the information of the recipient and clinical indication are stated. Immediate and short-term clinical effectiveness of implanted allograft are also reported. Any adverse reactions, the ease of handling, and any recommendations are to be filled in by the recipient center^9^.

## Human heart valve procurement and transplantation

### Heart valve replacement

Disease and congenital abnormalities can lead to defective heart valve function and cardiac insufficiency. Heart valve disease has devastating consequences and is burdensome for patients and health systems. Roughly 300,000 heart valve replacements are performed globally in a year^13,14^. All kinds of valve replacements come with an associated number of complications and drawbacks. In order to choose a suitable replacement among the available heart valve replacements, the advantages and disadvantages of each should be balanced and the patient’s general and clinical conditions must be taken into consideration.

Despite advances in autologous bioengineered valves, tissue (biological) and mechanical heart valve prostheses are currently and practically available. Biological valves can be xenografts (bioprostheses), or human tissue valve (homografts). Xenografts are typically constructed from either porcine aortic tissue or bovine pericardial tissue, and both are supported by a frame.

Recently, stentless (without frame) porcine valves have become widely used. The advantages of xenografts include a non-limited supply and better hemodynamics, compared to mechanical valves. Moreover, the risk of thrombogenicity in bioprosthetic valves is lower than with mechanical valves. However, progressive calcification and tissue deterioration shortens the durability of xenografts. Cryopreserved homografts, procured from deceased or living donors (from heart transplant recipients), thanks to their natural structure and morphology, give enhanced functional performance - and the presence of living cells is the reason for superior biocompatibility compared to other options.

Homografts have near-native hemodynamic properties, with resistance to infection and no need for long-term anticoagulant medication. The concern over homografts; however, lies in their inadequate durability (superior to xenogenic biological prostheses, but inferior to mechanical valves) and donor shortage^15–17^.

### Human heart valve procurement

 • Donor selection: In our current practice, potential donors are aged from 3 to 50 years old. In addition, diseased heart valves (congenital or acquired), endocarditis, and prolonged steroid use are conditions which rule these potential donors out.

• Tissue recovery: Recovery is performed in an operating theatre or a mortuary. Either a whole heart or heart block, observing aseptic condition and using sterile techniques (zone-specific prepping and draping), is retrieved within 24 hours of death, provided that the donor is refrigerated within 6-12 hours of death. A sample of tissue is taken as the first sample for culture. The retrieved tissue is washed with sterile isotonic solution (1-10° saline) and packed with Ringer solution in sterile double bags inside a plastic container and transported in wet ice conditions to the tissue bank, along with two 10 ml samples of the donor’s blood for microbiological and serological tests. In the processing unit, if immediate processing is not possible, the heart block is put into an antibiotic cocktail solution for disinfection at 4° for a maximum of 4 hours^10,18–20^.

• Donor screening: A meticulous donor screening system has to be implemented to assure that high quality tissues, with regards to safety and efficacy, will be recovered and processed. Physical examination, testing and autopsy are the main steps in screening. A fastidious and extensive donor screening process is primarily conducted to review the medical and social history of potential donors. Only positively assessed donors who meet the standards are accepted as actual tissue donors. This rigorous screening eliminates potential donors with even negligible risk of infection or malignancy. Since it is common for tissues from a single donor to be transplanted in more than 20 recipients, any risk associated with a donor will be amplified and could have enormous impact on disease transmission, although the overall risk of infectious disease transmission in tissue and cell recipients is much less than solid organ recipients, which in turn is less than 1%^21–23^. A Donor Risk Assessment Interview (DRAI), taking the donor’s medical, social and behavioral history, is the first step in donor screening to determine eligibility^12, 19, 24^. Long-term steroid use; diseases of unknown etiology such as sarcoidosis, Crohn’s disease, and ulcerative colitis; and multisystemic autoimmune diseases, like systemic lupus erythematosus and poly arthritis nodosa, are important to consider since these diseases by themselves, and their conventional treatments, may affect the quality of tissues^12^.

In physical examination, a close attention should be paid to evidence of non-medical injection sites (to exclude drug abusers), tattoos, white spots in the mouth, body piercing, scars, enlarged lymph nodes, incision sites, jaundice, genital lesions, open wounds, and hepatomegally^12^.

In testing, the most advanced testing equipment and techniques should be utilized. All conditions which may cause false positives or false negatives (massive transfusion, plasma dilution) must be taken into consideration^12,25,26^.

Hepatitis B Surface Antigen (HBS Ag), Hepatitis B Core Antibody (HBC Ab), Hepatitis C Virus Antibody and Nucleic Acid Testing (HCV Ab, NAT), Human Immunodeficiency Virus 1 and 2 (HIV 1,2), P24 Ag, Rapid Plasma Reagin (RPR), Veneral Disease Research Laboratory (VDRL), Human T-Lymphotrophic Virus 1 and 2 (HTLV 1,2), toxoplasmosis, Ebstein-Barr Virus (EBV), and Cytomegalovirus (CMV) are mandatory tests. It should be also mentioned that documents of eligibility of a donor must be retained for at least 10 years after the date of administration^25^.

• Tissue processing: The valve dissection is carried out under clean condition of class 100 laminar flow hood in a class 10,000 clean room. Clean room refers to a controlled environment where the concentration of airborne particles is controlled to specific limits, eliminating submicron airborne contamination. These contaminants (dusts, airborne microbes, aerosol particles) are generated by personnel, process facilities and equipment. The level to which these particles needs to be removed depends on the required standards. In class 100 and 10,000, the maximum allowable 0.5 micrometer particles per cubic foot are 100 and 10,000, respectively^27,28^.

Within 36 hours of asystole, and following cutting, physical cleaning (mechanical treatment) and trimming, heart valves are dissected and assessed from morphologic point of view. Valves are tested for competency, then ring size, length, and bifurcation length are measured and recorded. External and internal walls of the conduit are assessed in terms of atheroma, contusion, calcification and fibrosis. Cusps are also checked for fenestration, hemorrhage, fibrosis and atheroma. Morphological reasons and macroscopic lesions account for 3.1% of procured heart valves in our practice being rejected. Samples of the valves are also taken for second series of cultures.

All retrieved tissues (regardless of the 4th culture at the time of implantation) are cultured three times prior to storage, at the time of tissue recovery, and before and after antibiotic decontamination. Tissues are cultured in tryptic soy broth and thioglycollate broth to detect aerobic and anaerobic organisms, respectively. Sabouraud dextrose agar is also used for fungi cultivation. Two cultures in each medium are prepared and stored, one of them at room temperature and the second one at 37°C for 14 days, to detect slow growing organisms.

In case tissue culture results indicate the presence of virulent organisms such as Clostridia species, Entrococci, or fungi (first and second samples), the tissues are discarded. In our practice, the initial contamination rate of recovered heart valves (prior to antibiotic decontamination) is 36.8%. The valves are then treated with antibiotic solution which reduces the rate of contamination to 13.9%. In order to balance maximal viability, tissue integrity (observing optimal time for incubation) and lower rejection rate due to contamination, we adopted our new decontamination protocol since July 2014 as 100 microgram/ml Amikacin and 50 microgram/ml Vancomycin, and low dose 10 microgram/ml Amphotricin dissolved in M199 at 4° for 36 hours^29–31^.

• Preservation: The tissue cells must be viable for the maintenance of tissue function. Heart valves are preserved under cryogenic conditions. The valvular conduits are placed in an ice-cold cryoprotective solution. We use Medium 199 (M199) containing 5% human serum Albumin and 10% Dimethylsulfoxide (DMSO) as preservation solution. DMSO penetrates the cellular membrane and prevents ice crystal formation. A double sterile and heat sealed bag, covered by aluminium foil, is used to pack the heart valves immersed in preservation solution. The aluminium case permits appropriate heat exchange during freezing and thawing and prevents temperature gradients in the tissues. To efficiently preserve the matrix, the valves are cryoprerserved in a flat format to keep the leaflet position open^18,26,32–34^.

• Storage: The packed valves are then cryopreserved in a controlled-rate freezer at an initial rate of −1°C/min down to −60°C, then the second rate at −5°/min to −120°. After freezing, the valves are stored in the vapor phase of liquid nitrogen at −150°for up to 5 years. At this temperature, any biological activities which would cause cell death are prohibited. It is notable that allografts stored in liquid nitrogen for up to 13 years do not undergo any significant loss of cell viability other than that due to disinfection, freezing or thawing^8^.

• Release criteria: The most prevalent reasons why homografts failed to meet release criteria, in our practice, are positive microbiological culture and abnormal morphology - 13.9% and 3.1%, respectively (of a total 17% failure rate). The rate of successful implantation is 95% based on our current practice.

## Conclusion

Every year, more than 2 million patients worldwide gain benefit from the life-improving nature of tissue transplantation; however, there is an ever-widening gap between demand and supply. Tissue procurement intended for transplantation is a complicated but feasible activity in different settings with different levels of national income and infrastructure. Contrary to organ transplantation, which relies mainly on brain-dead donors, tissue donors are mostly deceased donors with circulatory death. As a result, donor shortage is not a concern. Among frequently used allografts, homograft heart valves are still the replacement of choice due to their near-native hemodynamic performance and lower incidence rate of complications, thanks to strict adherence to guidelines and regulations which have been developed over the years^35^.
